# Altered Volume, Morphology and Composition of the Pancreas in Type 2 Diabetes

**DOI:** 10.1371/journal.pone.0126825

**Published:** 2015-05-07

**Authors:** Mavin Macauley, Katie Percival, Peter E. Thelwall, Kieren G. Hollingsworth, Roy Taylor

**Affiliations:** 1 Newcastle Magnetic Resonance Centre, Institute of Cellular Medicine, Newcastle University, Newcastle upon Tyne, United Kingdom; 2 Medical School, Newcastle University, Newcastle upon Tyne, United Kingdom; Joslin Diabetes Center, Harvard Medical School, UNITED STATES

## Abstract

**Objective:**

Although impairment in pancreatic insulin secretion is known to precede the clinical diagnosis of type 2 diabetes by up to a decade, fasting blood glucose concentration only rises abnormally once the impairment reaches a critical threshold. Despite its centrality to the pathogenesis of type 2 diabetes, the pancreas is the least studied organ due to its inaccessible anatomical position. Previous ultrasound and CT studies have suggested a possible decrease in pancreatic volume in type 2 diabetes. However, ultrasound techniques are relatively insensitive while CT uses ionizing radiation, making these modalities unsuitable for precise, longitudinal studies designed to explore the underlying mechanisms of type 2 diabetes. Hence there is a need to develop a non-invasive, safe and precise method to quantitate pancreas volume.

**Methods:**

We developed and applied magnetic resonance imaging at 3.0T to obtain balanced turbo field echo (BTFE) structural images of the pancreas, together with 3-point Dixon images to quantify pancreatic triglyceride content. Pancreas volume, morphology and triglyceride content was quantified in a group of 41 subjects with well-controlled type 2 diabetes (HbA_1c_ ≤ 7.6%) taking only metformin (duration of T2DM 5.7±0.7years), and a control group of 14 normal glucose tolerance subjects matched for age, weight and sex.

**Results:**

The mean pancreatic volume was found to be 33% less in type 2 diabetes than in normal glucose tolerant subjects (55.5±2.8 vs. 82.6±4.8cm^3^; p<0.0001). Pancreas volume was positively correlated with HOMA-β in the type 2 diabetes subjects (r = 0.31; p = 0.03) and controls (r = 0.46; p = 0.05) considered separately; and in the whole population studied (r = 0.37; p = 0.003). In type 2 diabetes, the pancreas was typically involuted with a serrated border. Pancreatic triglyceride content was 23% greater (5.4±0.3 vs. 4.4±0.4%; p = 0.02) in the type 2 diabetes group.

**Conclusion:**

This study describes for the first time gross abnormalities of the pancreas in early type 2 diabetes and quantifies the decrease in pancreas size, the irregular morphology and increase in fat content.

## Introduction

Type 2 diabetes (T2DM) is caused by a combination of insulin resistance and decreased beta cell function [1, 2]. However, blood glucose levels do not rise unless pancreatic insulin secretory function has declined by approximately 50%, and the subsequent gradual deterioration of blood glucose control is related solely to declining beta cell competence [3–5]. Despite this, the pancreas remains the least studied organ in T2DM. This is principally a result of the inaccessible anatomical position of the organ and a lack of validated methods with which to undertake longitudinal studies. Although direct imaging of human beta cells is not yet possible, advanced magnetic resonance techniques to quantitate structure and chemical composition offer the possibility of defining the evolving pathophysiology within the pancreas in T2DM.

Three basic aspects of the pancreas may be of relevance to the development of T2DM. Firstly, the total volume of the pancreas must be considered given the known decrease in functional beta cell number as T2DM progresses [6, 7]. In T2DM, studies using ultrasound or CT have suggested a 7–22% decrease in pancreas volume [8–10]. Secondly, there has been no description of the overall appearance of the pancreas in T2DM. There is marked variation of pancreatic morphology in the general population, with a more serrated boundary of the pancreas generally ascribed to ageing [11–13]. Thirdly, the fat content of the pancreas has been reported to be related to beta cell function [14, 15]. However, measurement of pancreas fat using magnetic resonance spectroscopy has been questioned [16], and to avoid possible inclusion of signal from surrounding adipose tissue we developed an imaging-based method of high precision [17]. Using this method during rapid weight loss, the pancreas fat content has been shown to decrease over the same time course as return of normal insulin secretory capacity [17]. That longitudinal study was designed to evaluate the mechanisms underlying the return to normal glucose control during a very low calorie diet and was not large enough to quantitate precisely the difference in pancreatic volume and morphology between T2DM and age, weight and sex matched non-diabetic controls.

The present study was designed to define volume, morphology and fat content of the pancreas in a large group of individuals with T2DM.

## Methods

### Subjects

Forty-four subjects with well-controlled T2DM, (HbA_1c_ ≤ 7.6%) on metformin alone were recruited. This group was selected in view of the natural history of T2DM showing gradual need for additional oral agents with 50% requiring insulin therapy by 10 years after diagnosis [18], The mean duration of this group of T2DM with early stage disease thus defined was 5.7±0.7 years. A group of 14 normal glucose tolerance (NGT) subjects matched for age, weight and sex and with no first-degree family history of diabetes was studied to allow comparison. NGT was demonstrated in all control subjects by 75g oral glucose tolerance test (mean fasting plasma glucose 5.3mmol/l; 2h 5.5mmol/l). The clinical and metabolic characteristics are shown in Table 1.

**Table 1:** Anthropometry and metabolic characteristics of the Type 2 diabetes and Control groups.

	Type 2 diabetes (n = 41)	Controls (n = 14)	*p* value
Weight (kg)	87.2±2.0	86.7±3.7	0.81
BMI (kg/m^2^)	30.3±0.5	29.6±1.0	0.67
Body fat (%)	31.6±1.2	27.9±2.6	0.11
Waist/Hip ratio (-)	0.95±0.01	0.90±0.02	0.02
Age (years)	61.8±1.0	59.0±2.2	0.10
Fasting plasma glucose (mmol/l)	7.9±0.5	5.1±0.1	<0.0001
Fasting plasma insulin (mU/L)	13.3±1.3	7.9±0.9	0.002
HOMA-β (%)	72±7.1	97.3.0 ± 9.5	0.01
HOMA-IR (μU/mol/L^3^)	4.7±0.4	1.9 ± 0.2	0.02
HbA1c (%)	6.4±0.1	-	
Duration of diabetes (years)	5.7±0.7	-	

Data are shown as mean ± SEM

### Protocol

All subjects underwent metabolic and anthropometric characterisation prior to the magnetic resonance (MR) study. As the T2DM subjects subsequently took part in a randomized study of vildagliptin or placebo (n = 20 and n = 19 respectively; 2 did not complete due to unrelated medical problems), the opportunity was taken to re-study after 6 months of either vildagliptin plus metformin therapy or continued metformin therapy plus placebo. The outcome of that study and the liver triglyceride results shown (registered on ClinicalTrials.gov—NCT01356381) have been reported separately [19]. The study was approved by the Newcastle and North Tyneside 2 Research Ethics Committee (reference 13/NE/0208) and all subjects gave written consent.

### Pancreas volume quantification

All MR studies were performed on a Philips 3.0T Achieva scanner with a 6-channel cardiac surface coil used for signal detection (Philips Healthcare, Best, The Netherlands). A non-invasive magnetic resonance method was developed to quantify pancreas volume using balanced turbo field echo (BTFE) structural images. BTFE images contain a mix of T_1_ and T_2_ contrast, which distinguishes high signal intensity from vessels and visceral fat with lower intensity signals from the pancreas. It can therefore be used to clearly delineate the boundaries of the pancreas from the adjacent structures, including the surrounding visceral fat, the splenic vein, the superior mesenteric vessels, the inferior vena cava and the duodenum. Twelve axial sections of 5mm thickness were imaged during an eight second breath hold (repetition time/echo time/flip angle = 3.1ms/1.6ms/40°, field of view 400-480x300mm according to patient size, zero-filled to give resolution 1.39mm x 1.40mm, turbo factor 95, parallel imaging factor 2, bandwidth 1156 Hz per pixel) The polygon ROI tool in the freely-available imaging software Image J [20] was used to delineate the pancreatic tissue in each section. Integration over all the sections containing tissue allows us to obtain the total pancreas volume in cm^3^.

A pancreas volume index, defined as the pancreas volume in cm^3^ divided by the body surface area calculated by the method of Dubois and Dubois [21] was used to examine possible effects of different anthropometry on observed measurements [22]. The ratio of the pancreas volume to the waist circumference (in cm^3^/cm) was also calculated to evaluate the data corrected for potential excess visceral fat in T2DM subjects.

A Bland-Altman analysis was carried out to define repeatability and reproducibility of the pancreas volume measurement, with independent interobserver assessment of pancreas volume performed by two trained analysts (MM and KP). A subset of 20 randomly selected measurements were analysed twice by KP to define intraobserver statistics.

### Morphology

The anterior border of the pancreas was observed to be particularly variable in respect of degree of smoothness or irregularity. A five point semi-quantitative scale of irregularity (1 = most irregular, 5 = least irregular) with standardized outlines was used to score all pancreas scans, blind to T2DM/NGT status. Two observers (MM, RT) agreed the score for each pancreas in consensus.

### Pancreas and liver triglyceride quantification

Pancreas and liver triglyceride content were measured using the 3-point Dixon method [23] where three gradient-echo scans are acquired with adjacent out-of-phase and in-phase echoes (repetition time /echo times/averages/flip angle = 50ms/3.45, 4.60, 5.75ms/1/5°, bandwidth 435Hz/pixel). The fields-of-view and reconstructed resolutions were prescribed as per the BTFE sequence. 12 sections of 10mm thickness were used to image the liver, and 12 sections of 5mm thickness to image the pancreas, during four 17-second breath-holds. Custom MATLAB software was used to separate the fat and water contributions of the MRI and construct fat fraction maps. The polygon tool in the imaging software Image J [20] was used to define regions of interest within the homogenous pancreas parenchyma on both water images on three central sections of pancreatic tissue. For the liver, regions of interest on five homogeneous sections within the right lobe of the liver were evaluated, avoiding the gallbladder, large vessels and surrounding visceral tissue. The triglyceride content in the images was expressed as a percentage of the total signal from fat and water. The measurements from the defined sections were averaged.

### Body composition and anthropometry

A bioelectrical impedance device (Bodystat 1500, Bodystat Ltd, Douglas, UK) was used to measure percentage body fat. Waist hip circumference was measured with a tape measure with subjects in a relaxed standing position. The waist was defined as the mid-point between the lower edge of the ribcage superiorly and the anterior superior iliac spine inferiorly. The hip circumference was taken to be at the level of the greater trochanter.

### Metabolite and hormone assays

The plasma glucose concentrations were measured with a Yellow Springs glucose analyser (YSI, Ohio, USA). HbA1c was measured by high performance liquid chromatography (Bio-rad). Plasma insulin concentrations were measured with Dako Insulin enzyme linked immunoabsorbent assay (DAKO; Denmark) using a spectrophotometric analyser. An estimate of beta cell function (HOMA-β) was calculated from the fasting plasma glucose and insulin concentrations according to the homeostatic model of Matthews et al. [24], where HOMA-β = [20 x insulin conc^n^ (mU/L)]/[glucose conc^n^ (mmol/L)].

### Data analysis

Data are expressed as mean±SEM. Statistics were performed using Graphpad Prism 6.0d software). The Bland-Altman statistic was used to examine inter- and intra-observer repeatability and reproducibility [25]. Student's two-tailed paired *t*-test or the Mann-Whitney test, depending upon normality of data distribution, was used to compare within group and between group changes at different time points. Spearman correlation was used to assess relationship between variables. The distribution of the pancreas outline scores was compared by the Mann-Whitney test. A significance level of p < 0.05 was adopted.

## Results

### Pancreas volume

**Fig 1:**
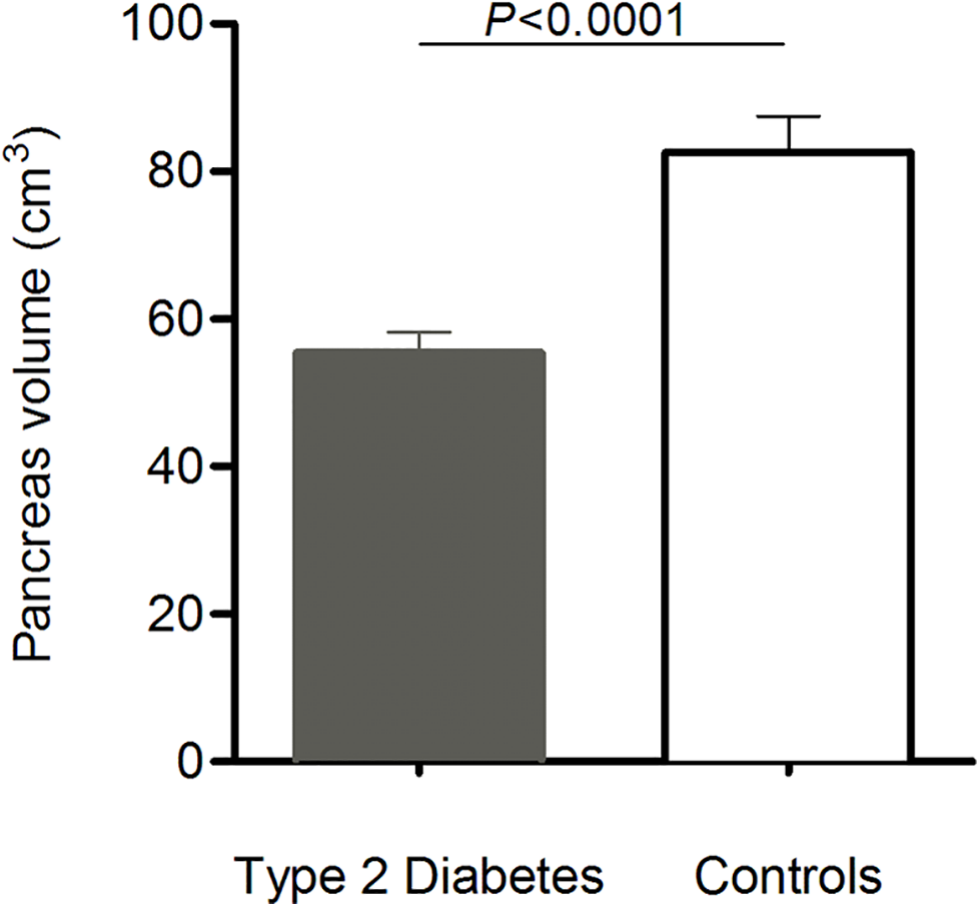
Mean pancreas volume in the type 2 diabetes and control groups (error bars show standard error of the mean). Pancreas volume was decreased by 33% (p<0.001 by Mann- Whitney U test).

Mean pancreas volume was 33% smaller in T2DM compared with NGT (55.5±2.8 vs. 82.6±4.8cm^3^; p<0.0001)(Fig 1). In T2DM, pancreas volume ranged from 20.8 to 99.7 cm^3^; and in NGT from 60.1 to 124.2 cm^3^. Expression of the data as pancreas volume index did not change the extent of difference between the groups (33%; 27.8±1.3 vs. 41.7±2.5cm^3^/m^2^; *p*<0.0001). When the data were expressed as ratio of pancreas volume to waist circumference, there was a 38% decrease in this ratio in T2DM subjects (0.53±0.03 cm^3^/cm vs. 0.85±0.06 cm^3^/cm; *p*<0.0001).

**Fig 2:**
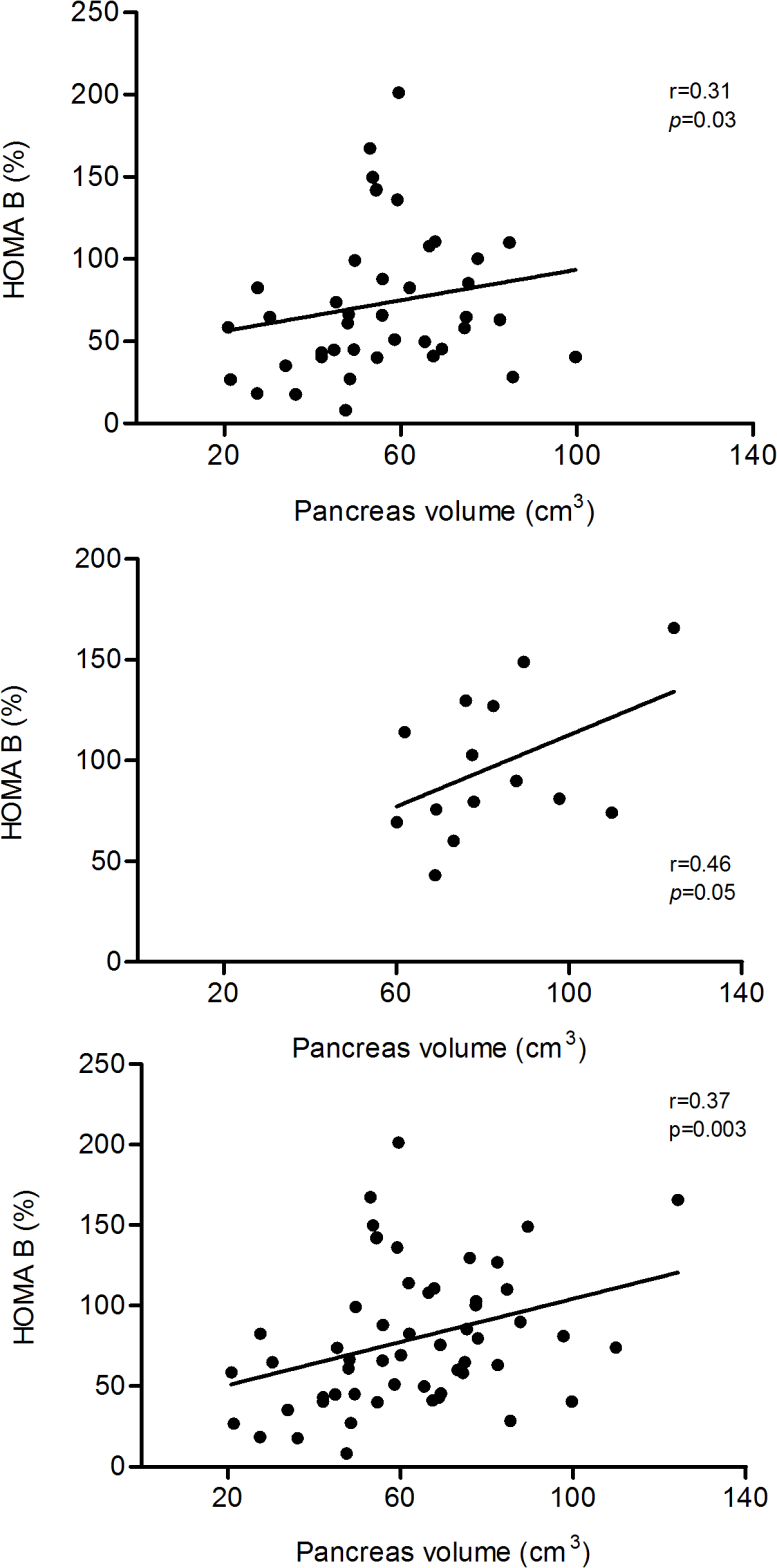
Relationships between HOMA-β (%) and pancreas volume (cm^3^) in T2DM group (panel a), control group (panel b) and all subjects combined (panel c). In each case there was a positive correlation between pancreas volume and insulin secretion (Spearman test).

There was a positive correlation between HOMA-β and pancreas volume in the T2DM group (r = 0.31; *p* = 0.03), the NGT group (r = 0.46; *p* = 0.05), and in the whole population studied (r = 0.37; *p* = 0.003) (Fig 2A–2C). Pancreas volume was inversely proportional to age in those under 60 years both in T2DM (n = 7; r = -0.56; *p* = 0.02) and in NGT (n = 6; r = -0.66; *p* = 0.09) and did not correlate with the duration of type 2 diabetes (r = -0.15; *p* = 0.19).

Pancreas volume remained constant over 6 months in both those T2DM subjects treated with placebo plus metformin (n = 19; 60.5±2.0 to 60.6±4.5cm^3^) and those treated with vildagliptin plus metformin (n = 20; 50.4±4.1 to 51.8±4.2cm^3^; *p* = 0.20).

**Fig 3:**
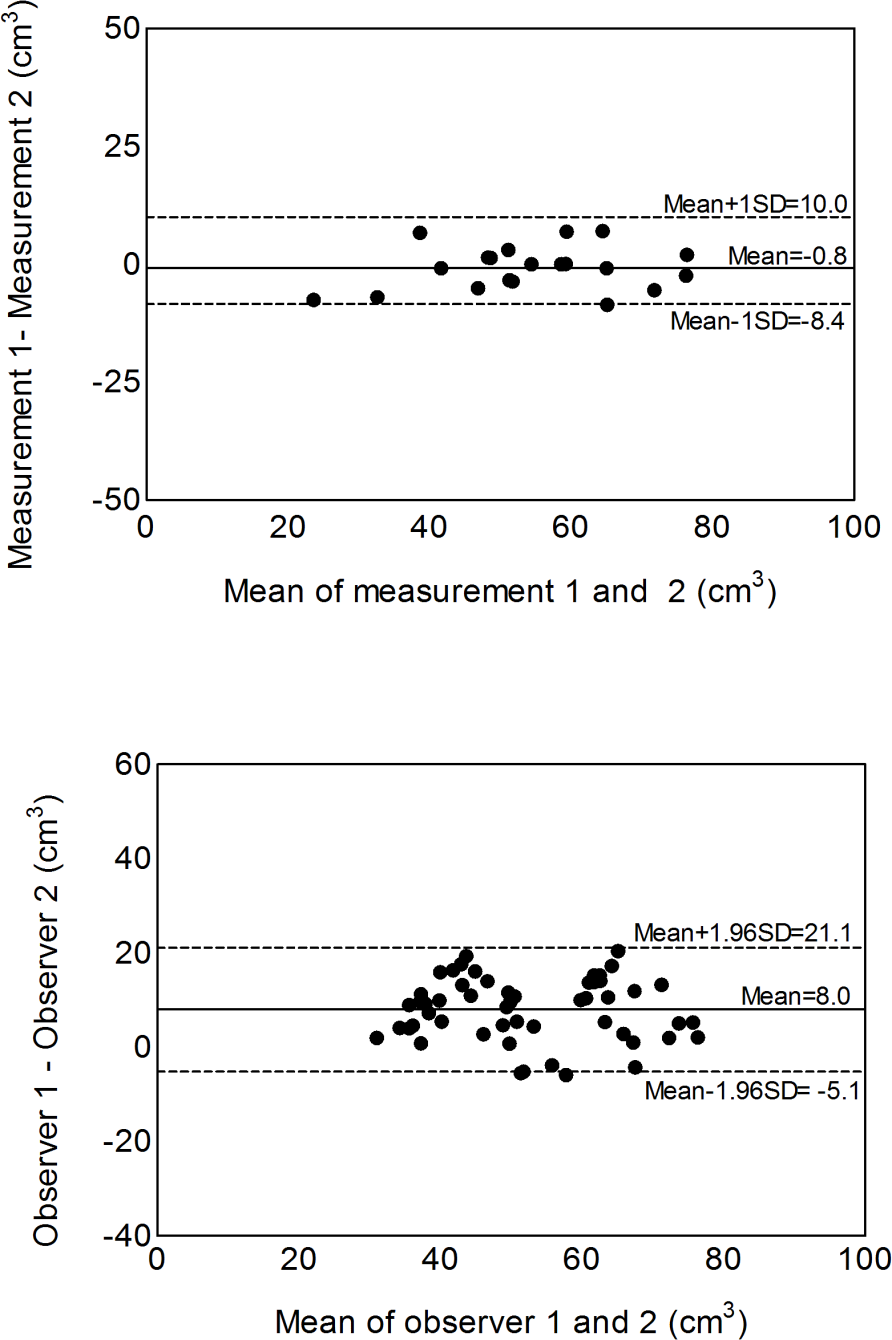
Bland-Altman plots for intra-observer (panel a) and inter-observer (panel b) analysis of pancreas volume measurement (cm^3^). The Bland Altman statistic confirmed lack of bias and defined 95% limits of agreeement.

The intra-observer bias of pancreas volume was 0.8cm^3^ with a 95% limit of agreement of 9.2cm^3^ (p>0.05) and inter-observer bias was 8cm^3^ with a 95% limit of agreement of 13.1cm^3^ (p>0.05) (Fig 3).

### Pancreas morphology

**Fig 4:**
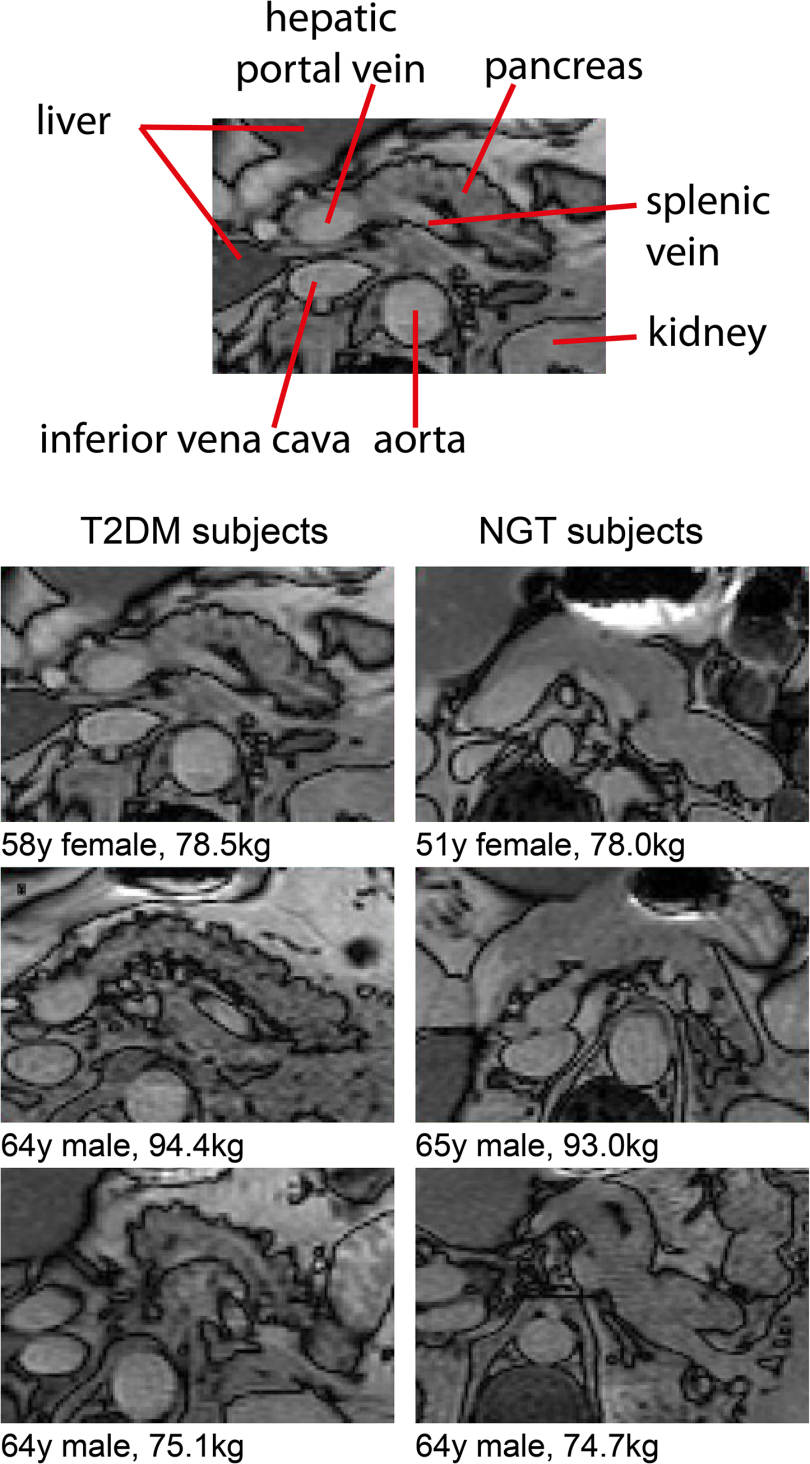
Comparison of the morphology of the pancreata of T2DM subjects (left) and NGT subjects (right) demonstrating the more irregular anterior (uppermost) border of the pancreata. In the upper panel anatomical landmarks around the pancreas have been labelledto allow interpretation of the comparative pancreas views below.

**Fig 5:**
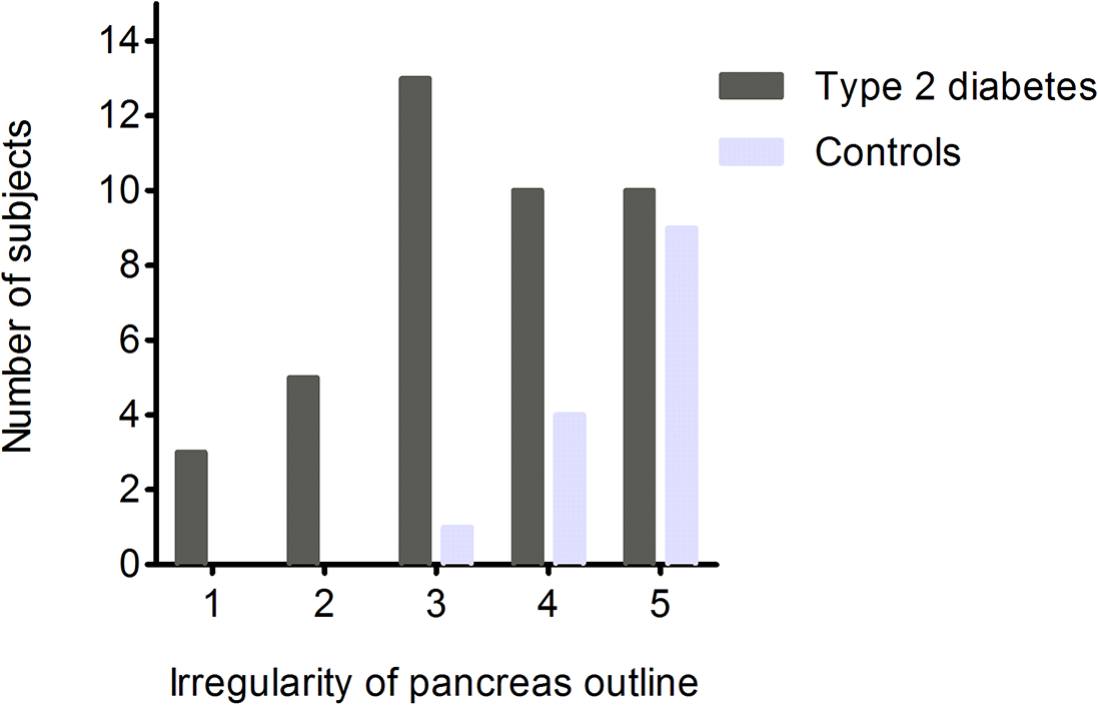
Semi-quantitive analysis of the irregularity of pancreas outline. Red: type 2 diabetes individuals; Green: non-diabetic control individuals. Most irregular = 1 and least irregular = 5. It can be seen that most non-diabetic individual have a smooth pancreas outline whereas many people with type 2 diabetes have an irregular pancreas border. The distributions of scores are significantly different (p<0.002; Mann-Whitney U test).

The pancreata of T2DM subjects were typically involuted, with a feathery, irregular border when compared with age, weight and sex matched glucose tolerant controls. This is illustrated in Fig 4. The semi-quantitative analysis of irregularity of the border of the pancreas is shown in Fig 5. Most controls were judged to have pancreata with regular, smooth outlines (modal score 5) whereas most T2DM subjects were deemed to have an irregular pancreatic outline (modal score 3). The distribution of scores was significantly different between T2DM and NGT subjects (p<0.002; Mann-Whitney U test).

### Pancreas triglyceride content

**Fig 6:**
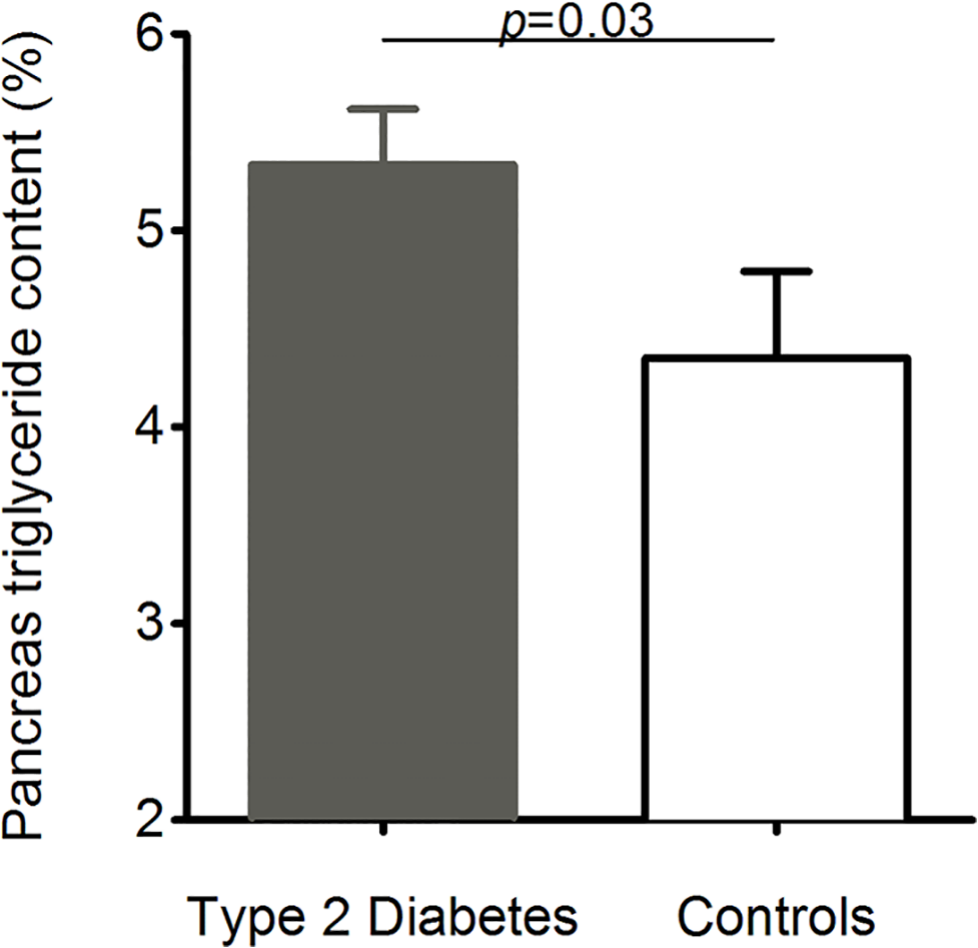
Pancreas triglyceride content in the T2DM and control group. The 23% higher triglyceride content in the T2DM group was significant (p = 0.02; Student’s t test).

Pancreas triglyceride content was 23% higher in T2DM compared with NGT (5.4±0.3 vs. 4.4±0.4%; p = 0.02)(Fig 6), while liver triglyceride content was 6.7±0.7% in T2DM and 3.4±0.7% in NGT (*p* = 0.0004) There was a positive correlation between pancreas triglyceride and BMI in NGT (r = 0.64; p = 0.008) and whole group (r = 0.33; *p* = 0.08), but this was not present in T2DM (r = 0.23; p = 0.08). There was a positive correlation between pancreas and liver triglyceride in the NGT (r = 0.63; p = 0.01) but this was lost in T2DM (r = 0.02; *p* = 0.46), and in the whole group (r = 0.18; *p* = 0.10). Liver triglyceride content has previously been reported to be (6.7±0.7 vs. 3.4±0.8%; P<0.001) [19].

Pancreas triglyceride content remained unchanged at 6 months during placebo and metformin treatment of T2DM (5.6±0.4 vs. 5.5±0.5ml; *p* = 0.90) and there was also no difference between the vildagliptin and metformin treated group (5.1±0.4 to 5.0±0.2%; *p* = 0.74).

## Discussion

This study demonstrates that the mean pancreatic volume is 33% smaller in individuals with type 2 diabetes well controlled on metformin compared to matched controls. Insulin secretion assessed by HOMA-β was proportional to pancreas size both in T2DM and NGT groups. For the first time the serrated boundary of the pancreas in T2DM is described. Not only is the pancreas small and involuted in appearance but also it has a 23% higher triglyceride content compared with that of well-matched non-diabetic individuals.

The question of cause and effect is immediately posed by these data. Are people with a small pancreas predisposed to develop T2DM, or does the pancreas decrease in volume as a consequence of the ongoing pathological processes? It has been postulated that beta cell mass may be subnormal in those predisposed to develop T2DM [26] but in the absence of a practical non-invasive method of measuring this *in vivo* it has not been possible to pursue this hypothesis. It is known that low birth weight is a risk factor for T2DM [27], and it may be considered that this could be associated with a smaller pancreas. If so, this could be interpreted as indirectly supporting the possibility of low pancreas volume and hence low beta cell mass as predisposing factors for T2DM.

However, the pancreas volume could secondarily be affected by loss of normal post-meal rise in insulin by a paracrine effect. Insulin potently stimulates growth and maintains tissue mass at around 10-fold greater concentrations than required for metabolic effects [28]. Pancreas tissue is likely to be exposed to very high concentrations of insulin after meals, when the local rise in concentration must vastly exceed the 10–15 fold rise achieved in plasma concentration [29]. When there is no local insulin production in type 1 diabetes, pancreas volume is known to be decreased by one third [30] and this is evident within months of diagnosis [31]. In T2DM basal insulin levels are raised but there is an absence of immediate and major insulin release after meals which could bring about growth promoting concentrations of the hormone. There are few animal studies of pancreas volume although a study in non-human primates observed a modest decrease in pancreas volume 2–6 months after inhibition of insulin secretion by low dose streptozotocin [32].

The data presently reported establish a significant relationship between pancreas volume and HOMA-β in both T2DM and non-diabetic groups. Further work is required to define the precise relationship of first phase insulin response to pancreas volume. We have previously demonstrated that acute insulin secretion in response to glucose and to arginine can be returned to normal in T2DM by substantial weight loss [17] and that normal glucose regulation is maintained for some years providing weight gain is avoided [33]. Studying larger groups of individuals with short or long duration T2DM has established that the condition can be reversed in most individuals up to 10 years duration [34]. It is now feasible to test the hypothesis that pancreas volume changes secondarily to development of the insulin secretory defect by studying prospectively a group of subjects with short to medium duration of type 2 diabetes over 6–12 months after restoration of normal insulin secretion and this work is underway.

Several previous studies have assessed the size of the pancreas in T2DM. A retrospective study of individuals who had undergone an abdominal CT scan reported a small decrease in pancreas volume in T2DM (7%; p<0.05) but the group was unusual in having a low BMI (mean 25.4) and body weight was not matched between T2DM and non-diabetic subjects [9]. Two ultrasound studies have examined pancreas cross sectional area in type 2 diabetes. One observed a significant 11% decrease compared with control subjects [8]. The larger ultrasound study of 84 T2DM and 80 controls matched for age, sex and body mass observed a 22% decrease [35]. The recent CT study of Korean subjects with mean BMI of 25.7kg/m^2^ and T2DM reported a 12–24% decrease in pancreas volume [10]. However, the abnormality has not been widely acknowledged or discussed. The present study used a method of defined precision in well matched groups to demonstrate a 33% decrease in pancreas volume in people with T2DM who were well controlled on metformin therapy alone.

The association of pancreas triglyceride with T2DM remains contentious. A cross-sectional study using ultrasound of 7,464 individuals showed higher pancreas triglyceride levels to be associated with obesity with both T2DM and pre-diabetes [36]. Magnetic resonance spectroscopy allows measurement of intra-organ triglyceride although it relies upon accurate respiratory triggering to acquire data solely from pancreatic tissue and not visceral fat. Using this method, it has been shown that higher pancreas triglyceride levels are associated with raised basal insulin levels, and that this relationship is present in different ethnic groups [14]. The wide scatter of absolute levels of pancreas triglyceride with a tendency for higher levels in people with diabetes has been confirmed [37]. The latter study showed that pancreas triglyceride correlated negatively with early glucose-stimulated insulin secretion. Because of the danger of incorporating visceral fat in the spectroscopic fat measurements, we developed an imaging-based method [17] that demonstrates higher precision than spectroscopic measurements. A longitudinal study of etiopathogenesis of T2DM using the new method has confirmed that weight-loss induced decrease in pancreatic triglyceride is temporally associated with restoration of normal glucose stimulated acute insulin secretion [17]. The present study has applied this method of magnetic resonance imaging to characterized individuals with T2DM with good glycemic control and treated only with metformin. We have demonstrated that pancreas triglyceride content is 23% higher than in age, weight and sex matched normal controls. Additionally, the data indicate that the normal positive correlation between pancreas and liver fat is lost in T2DM. This may be a consequence of falling plasma insulin levels during the development of T2DM [38]. This decrease would be expected to bring about decreased rates of *de novo* lipogenesis in the liver and potential loss of the initial relationship between liver and pancreatic triglyceride content. Further work is required to examine this possibility.

The limitations of the present study must be considered. Firstly, MRI measurement of organ volume has not previously been applied to the study of the pancreas in diabetes. The accuracy and precision of the method applied to pancreas require to be examined. Accuracy of such methodology compared to invasive tissue measurements is rarely established, but one study has shown volumetry by magnetic resonance imaging to correspond almost exactly to water displacement for pancreata of mini-pigs [39]. We demonstrate good precision as reflected by the Bland-Altman plots of intra- and inter-observer agreement. Additionally, repeat measurements after 6 months were highly consistent. Thus the measurement of pancreas volume by magnetic resonance imaging can be regarded as robust. Secondly, assessment of irregularity of shape of the pancreas can only be regarded as semi-quantitative and further work is required to develop a mathematical method for measuring deviation from a smooth but variable curve. Thirdly, accuracy and precision of pancreas triglyceride must be considered. Due to the rapid enzyme-mediated autolysis of the pancreas post-mortem, data on accuracy of human pancreas triglyceride measurement is lacking. However, we have previously reported an excellent inter-scan Bland-Altman coefficient [17] and the 3-point Dixon method used gives almost identical absolute measurements of intra-pancreatic triglyceride compared to those obtained by the centre with the largest experience of measurement using magnetic resonance spectroscopy [14]. Finally, the T2DM group was large for this type of detailed study. Although the group of subjects with proven NGT was smaller, the consistency of measured outcomes within the relatively homogenous control group allowed confident identification of the major abnormalities associated with T2DM.

In summary, this study has demonstrated that in a group of T2DM individuals with good metabolic control on metformin therapy alone the pancreas is 33% smaller in volume, is typically involuted and has raised triglyceride content compared to normal. These gross abnormalities could potentially yield prognostic information. Further work must elucidate their relationships with the pre-diabetes phase, their interaction with treatment modalities and changes during the natural history of type 2 diabetes.

## Supporting Information

S1 Data Individual data on pancreas volume, pancreas fat content and irregularity score for pancreas margin are listed for T2DM (sheet 1) and non-diabetic participants (sheet 2). (XLSX)
